# Bony Metastasis to the Radius as the Initial Presentation of a Lung Adenocarcinoma

**DOI:** 10.7759/cureus.36855

**Published:** 2023-03-29

**Authors:** Supun Gamage, Mohamed Zakee Mohamed Jiffry, Parathan Sriharan, Swarnakumar Velayuthum, Thanuka Gunawardana

**Affiliations:** 1 Orthopedic Surgery, National Hospital, Colombo, LKA; 2 Internal Medicine, Danbury Hospital, Danbury, USA

**Keywords:** palliative chemotherapy, lytic bone lesion, palliative radiation therapy, adenocarcinoma lung, bone metastasis

## Abstract

When considering tumors of the bone, metastatic disease from a distant primary is more common than primary tumors of the bone itself. The commonest sites to which skeletal metastasis occur are in the axial skeleton, and with regard to the appendicular skeleton, metastasis to the forearm bones is uncommon. Almost a third of patients who present with skeletal metastases do not have any evidence of their primary tumor at presentation. We report a case of a 68-year-old female diagnosed with lung adenocarcinoma after presenting with metastatic deposits involving the right radius as the first clinical manifestation of her disease. She presented initially complaining of painful swelling of her right forearm for a duration of one year. Imaging investigations of her right forearm showed an expansile mixed lytic and sclerotic lesion involving the full length of the right radius. A contrast-enhanced computed tomography scan of her chest to investigate the possible site of primary malignancy showed a peripherally located, well-defined, irregularly shaped mass lesion with enlarged mediastinal lymph nodes. A fluorodeoxyglucose positron emission tomography (FDG-PET) bone scan also noted oligometastatic disease in her right proximal humerus. She was started on palliative docetaxel for six cycles with palliative external beam radiotherapy. Although a variety of tumors metastasize to the bone, metastasis to the appendicular skeleton, and in particular the forearm bones, is a rare phenomenon that is poorly described in the existing literature. Skeletal metastasis may also be the primary presenting feature in a minority of cases. Lung cancer is among the more commonly associated primary sites, and further workup should include appropriate imaging to evaluate for a lung primary as well as an FDG-PET/CT or a bone scan to detect occult metastatic disease.

## Introduction

When considering tumors of the bone, metastatic disease from a distant primary is more common than primary tumors of the bone itself [1]. The most implicated primary tumor types prone to metastasis to the bone arise from the breast, lung, kidney, thyroid, and prostate. The commonest sites to which skeletal metastasis occur are the spine, ribs, ileum, sacrum, femur, humerus, scapula, and sternum [2].

With regard to the appendicular skeleton, up to two-thirds of these metastases are to the femur with much of the remainder being to the humerus, and metastasis to the forearm bones is uncommon [3]. These tumors can present with symptoms or signs of the primary tumor itself or of the secondary deposits, or even as paraneoplastic syndromes. The incidence of cancer of unknown primary (CUP) with skeletal metastases has declined over the years, with a 2012 study reporting an incidence rate of roughly 0.2 per 100,000 person-years [4]. Generally, such patients are considered to belong to an unfavorable subset if they have a CUP despite extensive diagnostic evaluation [5].

We report a case of lung adenocarcinoma presenting with metastatic deposits involving the full length of the radial bone as the first clinical manifestation of the disease.

## Case presentation

A 68-year-old female presented complaining of painful swelling on her right forearm for a one-year duration. The pain was of insidious onset and throbbing in character with no radiation. It was of moderate intensity and did not respond to analgesics. She had significant rest pain, pain at night, and pain that worsened with activity. It was not associated with evening pyrexia or other constitutional symptoms. She denied any history of trauma to the forearm. Her past medical history was not significant. She was a non-smoker with no history of occupational exposure to carcinogens. She had no personal or family history of malignancy. For her pain, she applied local applications of herbal treatment over her forearm, but decided to come to the hospital as her pain and swelling worsened over the last nine months.

**Figure 1:**
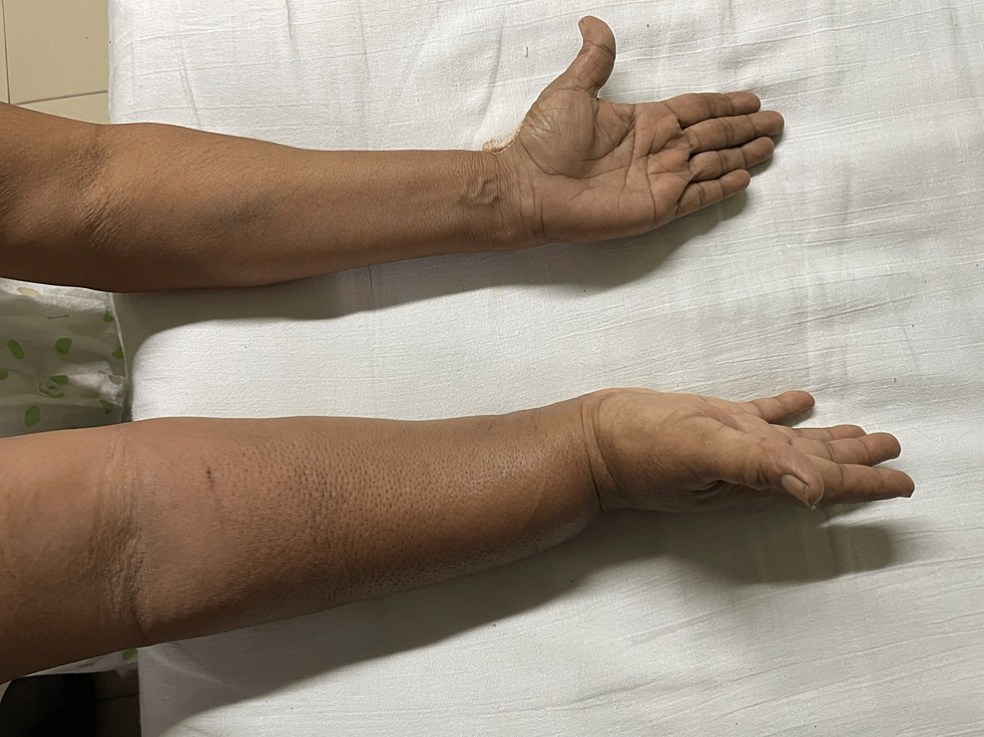
Comparative figure of the patient's left and right forearms showing edema of the right forearm with impaired supination.

On examination, she had right forearm pitting edema extending from the right elbow joint to the wrist joint (Figure 1). It was warm to the touch. The range of motion of her right elbow and wrist was restricted. No axillary lymphadenopathy was appreciated. Other system examinations including cardiorespiratory, breast, and thyroid were unremarkable.

Initial lab investigations included a complete blood count, which showed a normal white cell count. Her erythrocyte sedimentation rate (ESR) and C-reactive protein (CRP) were elevated (Table 1).

**Table 1:** Laboratory investigations obtained on day one of hospitalization. WBC: white blood cells; ESR: erythrocyte sedimentation rate; CRP: C-reactive protein; FBS: fasting blood sugar.

Lab investigation (units)	Day 1	Reference range
Complete blood count		
WBC (10^9^/L)	9.7	4.0-11.0
Platelets (10^9^/L)	455	150-400
Hemoglobin (g/dL)	11.8	11.8-14.8
ESR (mm/h)	80	<20
CRP (mg/dl)	65	<5
FBS (mg/dl)	107	70-115

**Figure 2:**
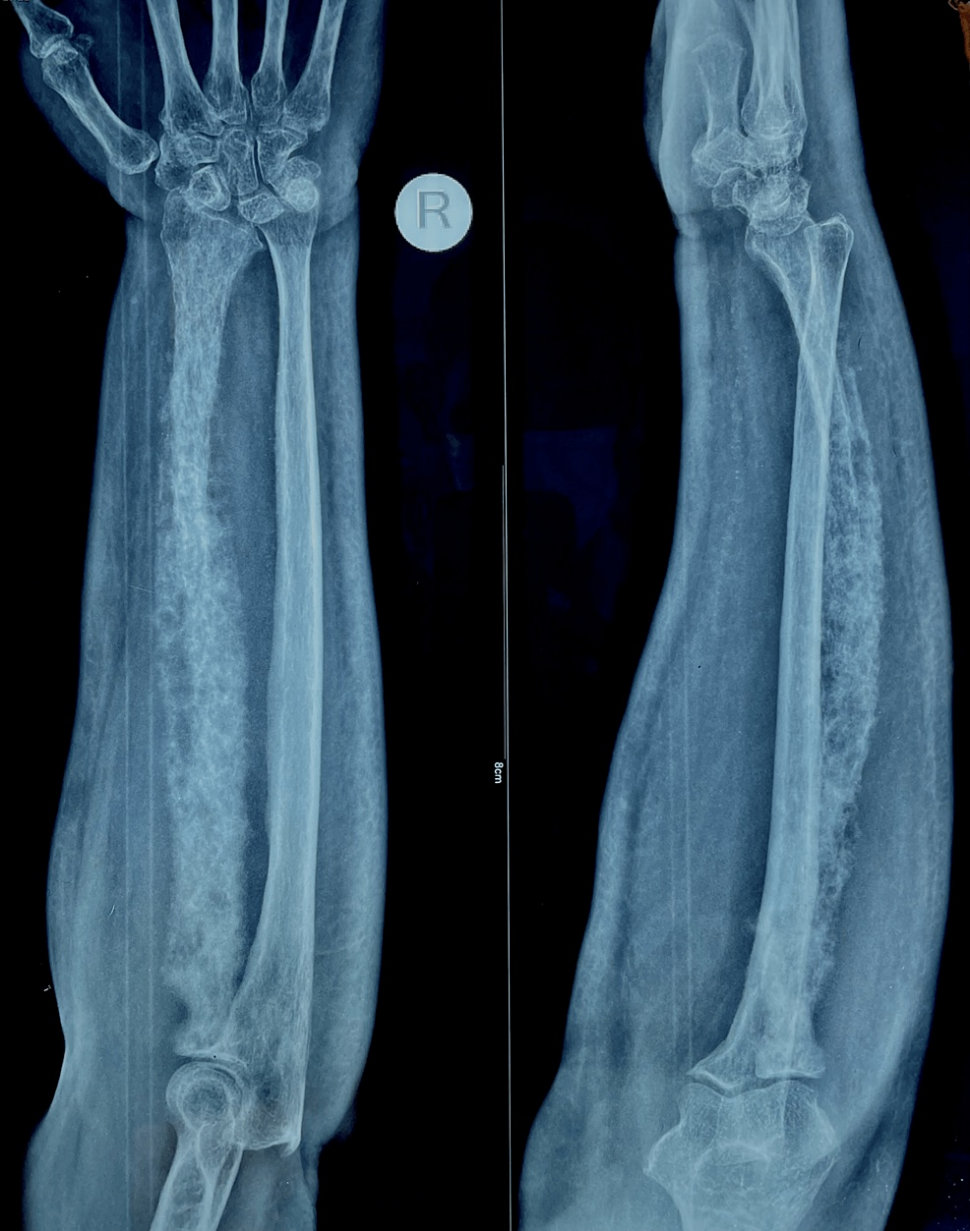
X-ray of right forearm showing an expansile mixed lytic and sclerotic lesion involving the full length of the radius.

Initial imaging investigations included an X-ray, ultrasound, and an MRI scan of the forearm. Forearm X-rays showed an expansile mixed lytic and sclerotic lesion involving the full length of the radius with an aggressive periosteal reaction and a pathological fracture in the distal diaphysis (Figure 2).

**Figure 3:**
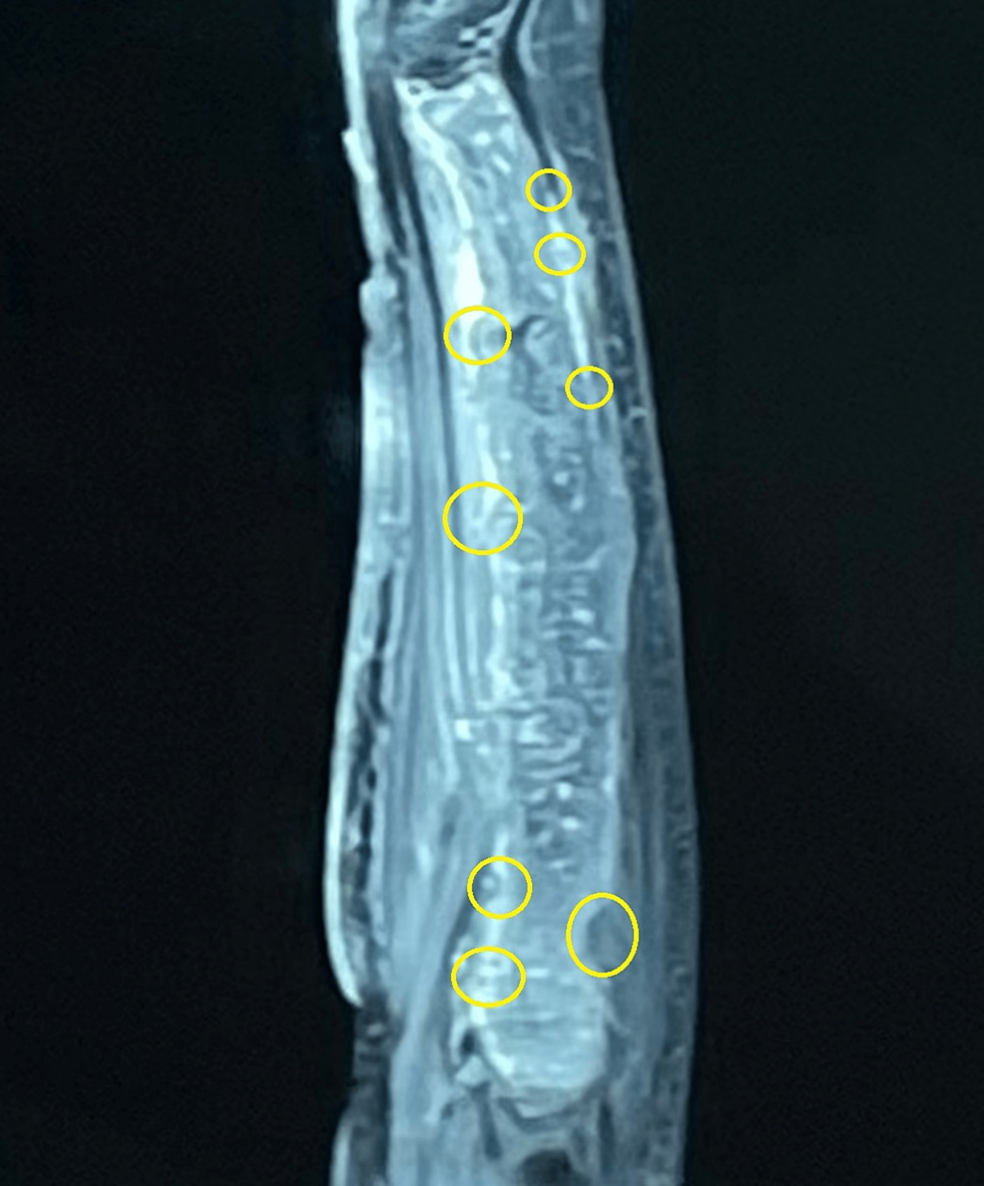
MRI of the right forearm showing cortical thickening and an irregular outline to the entire length of the radius. Yellow circles: Multiple ill-defined lytic areas on the inner aspect of the radius.

An ultrasound scan of the forearm showed diffusely thickened soft tissue of the right forearm with irregular outlines of the radius. Subperiosteal fluid was seen along the radius, although no evidence of a large collection was found. MRI of the forearm showed cortical thickening and an irregular outline of the radius with multiple small ill-defined lytic areas on the inner aspect. Minimal periosteal elevation and subperiosteal material were present in the region, and good but patchy contrast enhancement was present in the soft tissue component and the bone marrow (Figure 3).

**Figure 4:**
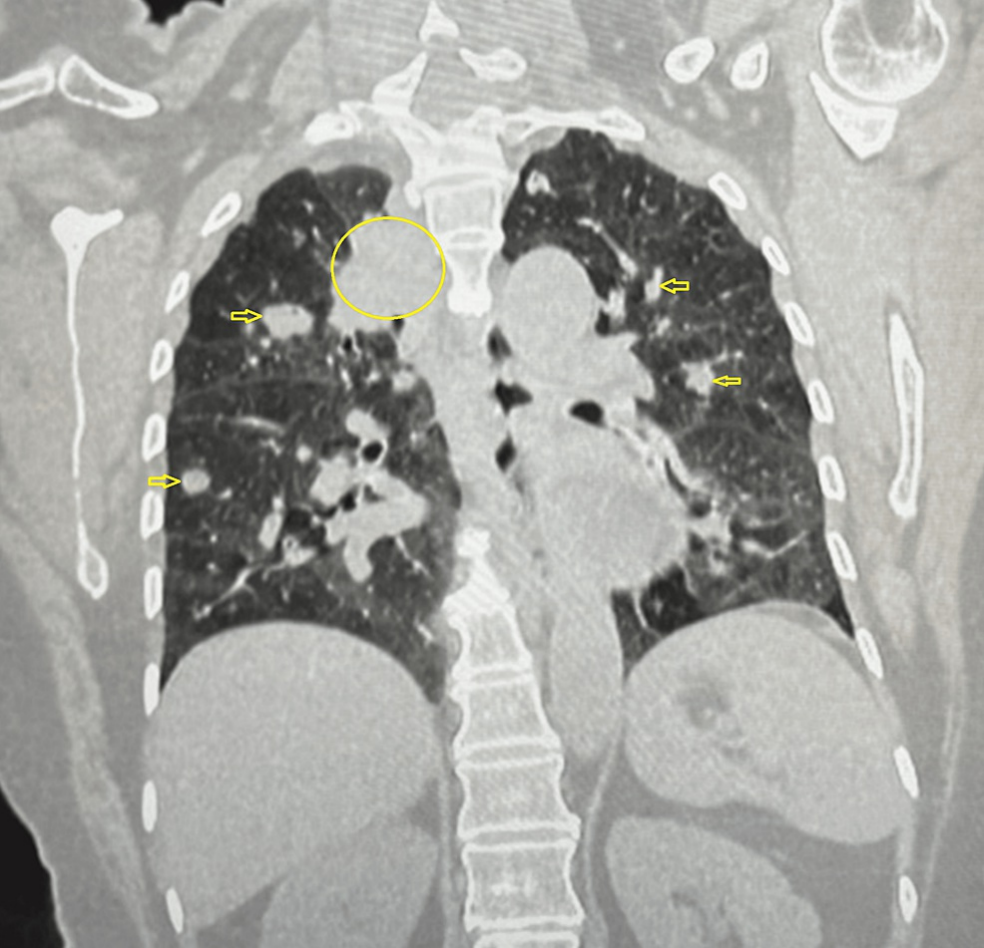
Contrast-enhanced computed tomography scan of the chest showing a large irregular apical mass lesion and multiple pulmonary metastases. Yellow circle: Irregularly shaped mass lesion in the apical segment of the right upper lobe measuring 33 x 30 x 40 mm in size. Yellow arrows: Multiple cavitary and non-cavitary pulmonary metastases.

Although her chronically progressive disease suggested a malignancy, chronic osteomyelitis was also a possibility given her imaging findings. A contrast-enhanced computed tomography (CECT) of the chest, abdomen, and pelvis to investigate the site of primary malignancy showed a peripherally located, well-defined, irregularly shaped mass lesion in the apical segment of the right upper lobe measuring 33 x 30 x 40 mm in size without evidence of local invasion. Multiple soft tissue density nodules of varying sizes were present bilaterally suggestive of multiple cavitary pulmonary metastases. Enlarged lymph nodes were present in the right and left lower paratracheal groups, the para-aortic group, and the subcarinal mediastinal group (Figure 4).

**Figure 5:**
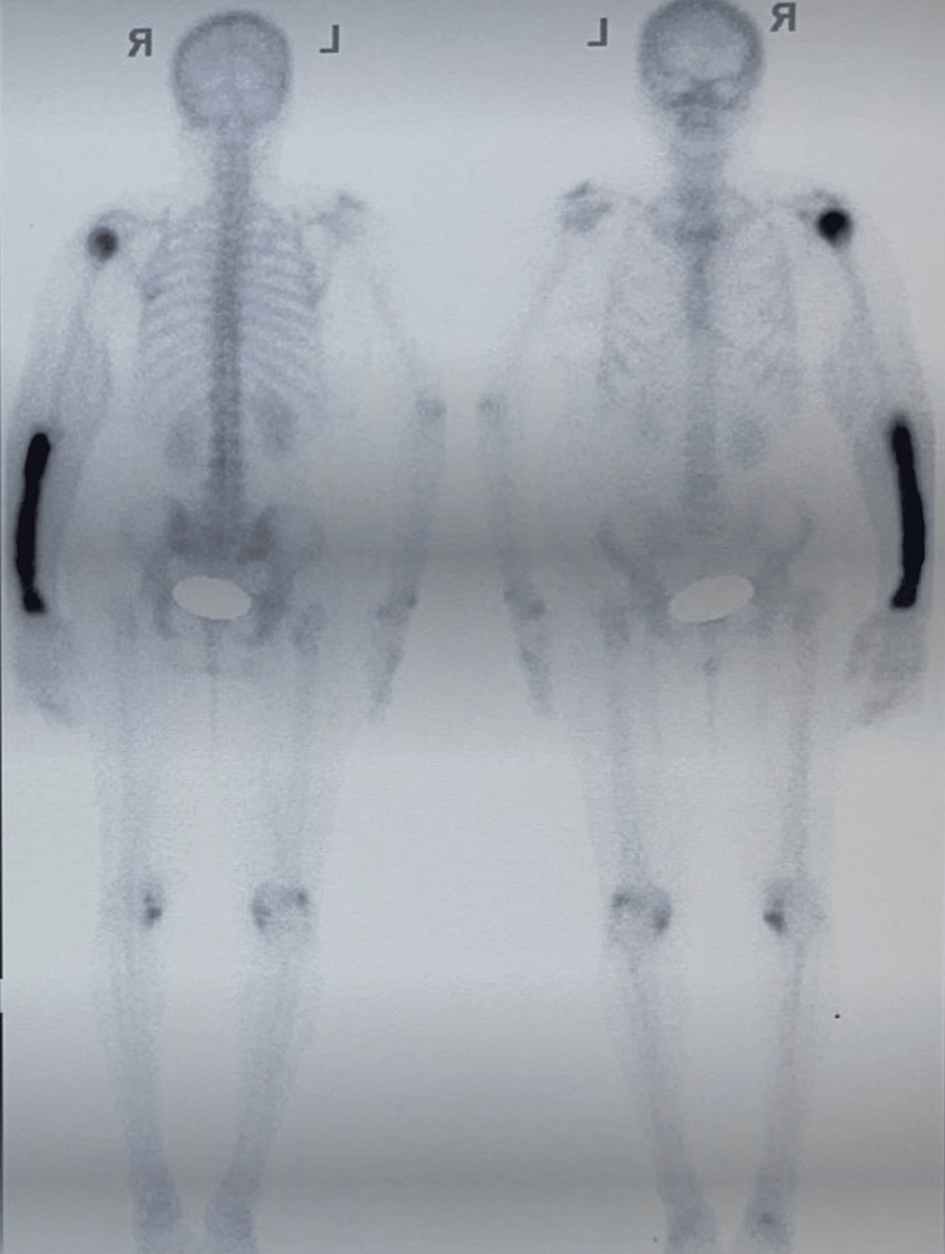
A technetium-99m bone scintigraphy study (bone scan) showing focal uniform tracer uptake in the right radius corresponding to the bony lesion as well as in the right proximal humerus suggestive of bone metastasis.

An ultrasound-guided biopsy of the right radius was performed, and subsequent histology and immunohistochemistry showed infiltrating cords of cells with enlarged, pleomorphic nuclei positive for cytokeratin 7 (CK7) and transcription termination factor 1 (TTF1). The overall immunomorphology was consistent with metastatic deposits from primary lung adenocarcinoma. An ultrasound scan of the neck and breast was unremarkable. A bone scan was performed to assess the extent of skeletal metastasis, which showed focal uniform tracer uptake in the right radius corresponding to the bony lesion. Increased uptake was also noted in the right proximal humerus suggestive of another metastatic bone lesion (Figure 5).

A multidisciplinary team meeting was held with oncology, orthopedic surgery, radiology, and histopathology to discuss the next steps. After further discussion with the patient and her family, it was decided to pursue palliative chemotherapy and radiotherapy. She is currently undergoing palliative chemotherapy with docetaxel for six cycles and awaiting palliative external beam radiotherapy of her forearm.

## Discussion

Bone is the most common overall site to be affected by distant metastases, as well as the site that produces the most morbidity [6]. A wide variety of solid tumors metastasize to the bone, with prostatic and breast cancers being the most commonly implicated. Other cancers that commonly metastasize to the bone include thyroid, kidney, and lung malignancies, with the incidence of bony metastases from lung malignancies being reported as high as 36% [7].

As previously mentioned, bone metastases most commonly affect the axial skeleton, and metastases to the appendicular skeleton distal to the elbow in the upper extremity and distal to the knee in the lower extremity are extremely rare. Interestingly, when malignancy in these regions is identified, the most common primary sites identified are renal and lung carcinoma [8]. Interestingly, our case presented chiefly with symptoms and signs of the secondary metastatic deposit as opposed to those of the primary lesion, and this holds true for up to 25-30% of cases of skeletal metastasis [9,10]. Very few existing reports detail skeletal metastasis to the forearm bones, and unilateral metastasis as seen in our case is even more uncommon [11].

There is no standard approach regarding the workup of a detected osseous metastasis in a patient with suspected cancer. Investigations focused on finding a likely primary may be guided by the radiographic presentation. For example, predominantly osteoblastic metastases are more likely in cancers such as prostate, carcinoid, and Hodgkin’s lymphoma. Peripheral bone metastases from lung cancer are virtually always osteolytic lesions, which is consistent with the imaging findings in our case [8]. MRI and CT imaging of affected extremities can help delineate the extent of involvement as well as assess for concomitant fractures and extraosseous soft tissue extension. A fluorodeoxyglucose positron emission tomography (FDG-PET) scan is also recommended for further evaluation of primarily lytic tumors, as it is a more sensitive modality for the evaluation of bone metastases than plain radiography [12].

In general, age alone should not be a barrier to systemic therapy in the management of advanced non-small cell lung cancer as they have been found to benefit from such therapy to the same degree as younger patients, although other factors such as medical comorbidities and patient preference, as in our case, do play a significant role. The efficacy of docetaxel as single-agent therapy was demonstrated in a trial of 104 patients with advanced non-small cell lung cancer as compared to best supportive care alone, with patients who received docetaxel having had a significantly longer overall survival (median of 7.5 vs. 4.6 months). They also had improved pain control and significantly less deterioration in their quality of life [13]. External beam radiation therapy is also effective in partially or completely relieving pain in a majority of patients with bone metastases and is therefore recommended for symptomatic relief [14].

## Conclusions

Although a variety of tumors metastasize to the bone, metastasis to the appendicular skeleton, and in particular the forearm bones, is a rare phenomenon that is poorly described in the existing literature. Skeletal metastasis may also be the primary presenting feature in a minority of cases. Lung cancer is among the more commonly associated primary sites, and further workup should include appropriate imaging to evaluate for a lung primary as well as an FDG-PET scan to detect occult metastatic disease.
